# Deformation Monitoring for Chinese Traditional Timber Buildings Using Fiber Bragg Grating Sensors

**DOI:** 10.3390/s18061968

**Published:** 2018-06-19

**Authors:** Ni-Lei Li, Shao-Fei Jiang, Ming-Hao Wu, Sheng Shen, Ying Zhang

**Affiliations:** 1 College of Civil Engineering, Fuzhou University, Fuzhou 350108, China; linilei@foxmail.com (N.-L.L.); clevermouse11@163.com (M.-H.W.); S_Shen@126.com (S.S.); 2 College of Architecture, Fuzhou University, Fuzhou 350108, China; zy6163@163.com

**Keywords:** deformation monitoring, FBG sensing, mortise-tenon joint, inclination angle, amount of dislocation, semi-rigid joint, Chinese traditional timber buildings

## Abstract

The Fiber Bragg Grating (FBG) sensing technique is suitable for a wide variety of measurements, including temperature, pressure, acceleration, liquid level, etc., and has been applied to many bridges and buildings in the past two decades. The fact that the FBG technique can only monitor and measure strain data for most cases when it is used for deformation measurements impedes application of the FBG sensing technique in civil infrastructures. This paper proposes FBG sensing-based deformation monitoring methods that are applicable to monitoring beam deflection, column inclination angle and mortise-tenon joint dislocation for Chinese traditional timber structures. On the basis of improved conjugated beam theory and geometrical trigonometric function relationship, the relationships between the FBG sensing strain values and the deflection of beam, inclination angle of column, as well as the amount of dislocation of mortise-tenon joint are deducted for Chinese traditional buildings. A series of experiments were conducted to verify the applicability and effectiveness of the proposed deformation monitoring methods. The results show that a good agreement is obtained between the values given by the methods proposed in this paper and other methods. This implies that the proposed deformation monitoring methods are applicable and effective in the health monitoring of Chinese traditional timber structures.

## 1. Introduction

There exist a huge number of ancient timber buildings and modern newly-built timber houses in China. These timber buildings have suffered more or less serious damage due to historical changes, long-term loads and environmental effects, which may lead to cumulative damage and even structural collapse [1,2,3,4,5,6,7]. To maintain and protect these historical and cultural heritage buildings, and assure their safe operation, it is becoming increasingly important and urgent to monitor their daily state so as to provide alarming prior to the occurrence of damage or failure. To solve such a problem, the current timber buildings design specification of China [8] requires timely observation in either of the following cases: (1) Inclination, slanting, or twisting ever occurring in the buildings, or a slow development of these caused by uneven settlement; and (2) load-bearing members displaying significant flexural cracking or a large amount of dislocation in mortise-tenon joints. As a consequence, to develop effective and practical deformation monitoring methods for timber buildings, it is a really significant task.

A large number of deformation monitoring and/or detecting methods and instruments have been developed and applied to bridges, dams and buildings, such as linear variable differential transducers (LVDTs), inclinometers and strain gauges. In recent years, with increasing research on novel and smart sensors [9,10,11], many scholars have gradually applied these sensors and sensing technologies, like novel giant magnetostrictive actuators, PVDF thin-film transducers, piezoceramic transducers, fiber Bragg grating (FBG) sensors, to the field of civil engineering [12,13,14,15,16,17]. Actually, many of these sensing and monitoring methods have been gradually adopted to detect the deflection, inclination angle and strain for timber bridges and buildings [1,2,3,4,5,6,7,8,18,19]. Some new monitoring techniques were developed especially for museums and historic cultural heritage buildings [4,19,20,21]; furthermore, new techniques were employed to develop structural health monitoring (SHM) systems for the purpose of protecting historic cultural heritage and buildings [2,3]. For example, Feio et al. [19] reviewed the application of visual strength grading standards in situ and the way the information could be combined with information provided by other nondestructive testing/semi-destructive testing methods to obtain a more reliable assessment of the mechanical performance of timber structural members. Due to the superior virtues of the FBG sensing technique, Marsili et al. [21] proposed the use of FBG sensors to monitor timber beams under operating conditions and to analyze the effects of structural reinforcements applied on them. In view of the fact that SHM systems can provide enough information regarding the structural performance and have been widely used on large-scale or complicated structures, Wang et al. [22] developed a SHM system frame and structure for heritage buildings, and then the feasibility of the application was validated by an experiment on a Tibetan heritage building; finally, a perspective on SHM development trends in the future was given. There is no doubt that these studies have all contributed to understanding the basic principles of FBG techniques and their application in timber buildings. However, Chinese traditional timber buildings typically have special construction features like mortise-tenon joints, which result in unique mechanical properties [4]. It is not suitable for Chinese traditional timber buildings to adopt the commonly used deformation transformation algorithm with the FBG strain data [20,23], which can cause large measurement errors and negative influence on the safety assessment and accuracy of the traditional timber buildings.

To solve the abovementioned problem, the authors [18] presented a deformation monitoring method for traditional wooden beams based on FBG strain data and a mathematical algorithm, and the efficiency of the method was validated on the beams of a wooden-frame structure. As the extension of the presented method [18], this paper also aims to develop monitoring methods of beam deflection, column inclination angle and mortise-tenon joint dislocation for Chinese traditional timber buildings using FBG sensors. Consequently, the rest of this paper is organized as follows: Section 2 proposed three deformation monitoring methods for different types of members based on FBG sensors. A series of experiments were conducted to verify the effectiveness of the proposed deformation monitoring methods in Section 3. Conclusions and remarks are given in Section 4.

## 2. Deformation Monitoring Methods Based on FBG Sensors

According to the abovementioned discussion, measurement sensors should meet the requirements of long-term monitoring, small space occupation and easy installation with low disturbance to protect precious historical cultural treasures as much as we can.

Compared with electrical sensors, FBG sensors are free from the electromagnetic interference and time-related drift typically encountered with electrical sensors. Owing to its protecting sleeve, a FBG sensor can work well for a long period of time in a harsh environment, which means it can be used in real-time safety monitoring outdoors. Moreover, the FBG has traits of light weight, small size, and can be bonded directly on the surfaces of the structures without using any nails and bolts, which meets the need of low disturbance to the original structure and small space occupation in the ancient timber structures to be monitored. This paper develops methods to convert the micro-deformation-strain measured by FBG to the macro-deformation, of our concern, i.e., deflection of beam and inclination of column. This is the so called FBG sensing technique.

### 2.1. Deflection Monitoring of Chinese Traditional Timber Beams

An analytical method of beam deflection was deduced on the basis of the strain reading with FBG sensors, taking into consideration the following three features for Chinese traditional timber buildings:

- Semi-rigid joint support
- Support settlement
- Irregular section

The applicability of the proposed method was also discussed in this paper, taking into account the span-depth ratio and the number of measurement points.

#### 2.1.1. Strain-based Deflection Equation Derivation for Simply-Supported Beam

When a simply-supported beam suffers from applied loads, the relationship between strain distribution on the cross section of the beam and the applied loads can be illustrated in Equation (1) according to the Euler beam assumption:

(1) $$\Delta \varepsilon (x)=-\frac{M(x)}{EI}\cdot \Delta z(x)$$

where x notes the location of a cross section; M(x), Δε(x) are the moment and strain distribution in the x-direction of the beam, respectively; EI and Δz(x) indicate the section stiffness and distance from sensor location to inertial axis, respectively.

An approximate differential equation of beam deflection can be obtained by the fundamental theory of material mechanicals [24] and is shown in Equation (2):

(2) $$\frac{d^{2}w(x)}{d^{2}x}=-\frac{M(x)}{EI}$$

where w(x) is the deflection function of timber beam.

After combining with Equations (1) and (2) and performing two integrations, it’s not difficult to obtain the relationship between deflection and strain distribution as shown in Equation (3):

(3) $$w=\iint \frac{\Delta \varepsilon (x)}{\Delta z}dxdx,$$

To calculate the deflection at a certain point the beam should be divided into *n* equal length units on the basis of conjugate beam theory [23]. So, the Equation (3) can be transferred as Equation (4):

(4) $$w_{p}={\left(\Delta l\right)}^{2}\left[\frac{p}{n}\sum_{i=1}^{n}\left(n-i+\frac{1}{2})(\frac{\varepsilon_{i}}{z_{i}}\right)-\sum_{i=1}^{p}\left(p-i+\frac{1}{2})(\frac{\varepsilon_{i}}{z_{i}}\right)\right],$$

where, *w_p_* is the deflection deformation value at the demarcation points between unit *p* and *p* + 1; *n* is the total number of beam elements divided; *i* is the number of unit, *i* = 1, 2, …, *n*; $\varepsilon_{i}$ is the average strain difference between the upper and lower surfaces of each beam element; $z_{i}$ is the beam section height; Δl is the length of each unit.

#### 2.1.2. The Influence Factor Analysis

Different from concrete or steel simple-supported beams, the Chinese traditional timber beam must take three factors into consideration, namely the semi-rigid tenon joint support, support settlement and irregular section.

##### Semi-Rigid Tenon Joint Support

**Figure 1:**
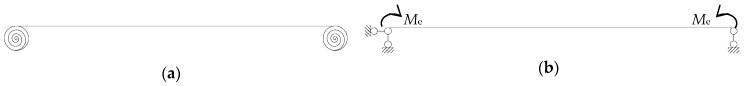
Calculation diagram: (**a**) a timber beam with semis-rigid support; (**b**) a simply supported beam with extra moment.

Chinese traditional timber beams and columns are connected by tenon joints, and thus both ends of the wooden beams are semi-rigid supports, whose constraints fall between those of simply-supported beams and fixed beams. That is to say, the semi-rigid support has a certain bending moment when suffering the applied loads or support settlement. Therefore, Chinese traditional timber beams can be considered equivalent to a simply supported beam with an equivalent bending moment $M_{e}$ at the end of the support, as illustrated in Figure 1.

The value of $M_{e}$ depends on the member size, applied loads and magnitude of support settlement. When load is applied on the beam or non-uniform support settlement there occurs, the total strain Δε of the timber beam shown in Equation (3) can be depicted as Equation (5):

(5) $$\Delta \varepsilon =\Delta \varepsilon_{F}+\Delta \varepsilon_{M}$$

where Δε is the total strain, $\Delta \varepsilon_{F}$ is the strain caused by the applied load, and $\Delta \varepsilon_{M}$ is the strain induced by the equivalent additional bending moment of the semi-rigid support.

##### Support Settlement

Different from a simply-supported beam, the non-uniform support settlement will lead to additional equivalent bending moments for a Chinese traditional timber beam, depicted as $\Delta \varepsilon_{M}$ in Equation (5), which will change the strain of a timber beam and thus affect the deflection. Consequently, the effect of the support settlement only needs to be linearly superimposed on the Equation (3), and the linearity is superposed according to the ratios of the support settlement values Δ*l*, Δ*r* at the left and right ends. The final total deflection $w_{T}$ is shown in the following Equation (6):

(6) $$w_{T}=w+w_{s}=\iint \frac{\Delta \varepsilon (x)}{\Delta z(x)}dxdx+\frac{l-x}{l}\Delta_{l}+\frac{x}{l}\Delta_{r}$$

where, deflection $w_{T}$ is the total deflection; w is the deflection for a simply-supported timber beam according to Equation (4); $w_{s}$ is the deflection induced by the rigid body displacement, namely support settlement; l is the length of the simple beam.

##### Irregular Section

**Figure 2:**
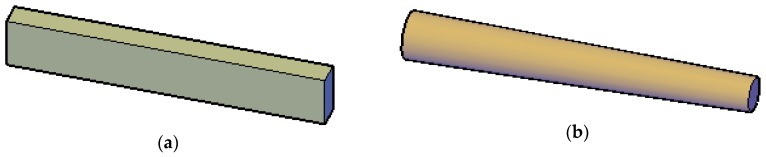
(**a**) concrete beam; (**b**) log beam.

Different from concrete or steel beams, the section of log beams is irregular, as shown in Figure 2.

Even if the cross sections are different along the length of a log beam, Equation (6) is still applicable because of the small deformation hypothesis. Besides, in Equation (6), Δz is the section height of each measurement location instead of the average section height, in which way, irregular sections of the log beam have been reflected in the algorithm.

#### 2.1.3. Feasibility of the Timber Deflection Equation

Obviously, the accuracy of deflection calculation depends on the following two factors: span-to-height ratio, *k*, which decides whether or not to consider shear deformation; and the total number of beam elements divided, *n*, namely the number of FBG sensors.

The above deduction is based on the Euler beam hypothesis. The larger the span-to-height ratio is, the more accurate the result is. The span-to-height ratio *k* of the load-bearing purlins and grids of Chinese traditional timber structures range from 20 to 40 [25]. At a low span-to-height ratio (*k* ≤ 10), the forehead beam lifting joints does not undergo excessive deformation, thus the timber beam with a span-to-height ratio of 20–40 is discussed.

**Figure 3:**
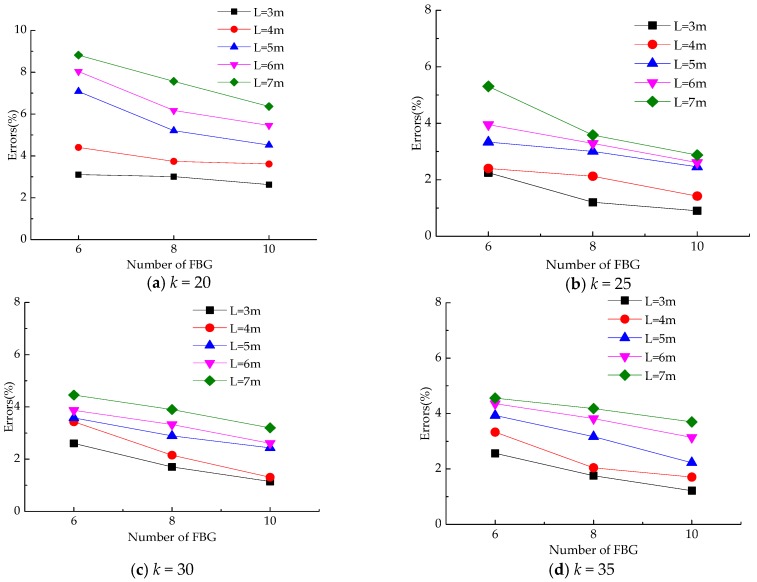

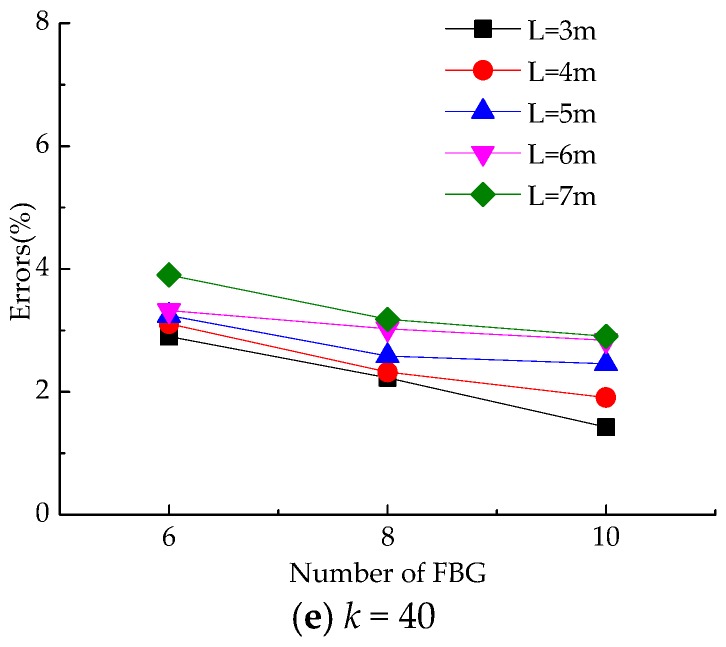
The relationship between the calculation error and the number of measurement points for different span lengths and span-to-height ratios.

In addition, the width and length of the general in-service timber dwelling are about 3–5 m [26]. Taking into account slightly larger width of the building, a timber beam with the span of 3–7 m is used for determination of the elements number. ANSYS is used to establish a single-span timber frame simulation analysis model, in which the differences in calculation errors between different span-to-height ratios and the number of partitioned units (the number of sensor measurement points) are compared. The errors between calculated values and measurement results are shown in Figure 3. The calculated value is obtained by Equation (6) and the measurement results are taken from the finite element model. From the above figures, the following conclusions can be drawn: When *k* is constant, the greater the span length is, the more the sensor measurement points are. At the same time, when the span length is constant, the smaller the span-to-height ratio of the beam is, the more the number of FBG sensors is. As a result, the optimum number of measurement points is recommended for actual monitoring as shown in Table 1.

### 2.2. Tilt Monitoring of Chinese Traditional Timber Column

#### 2.2.1. FBG-Based Tilting Monitoring Method of Timber Column

**Figure 4:**
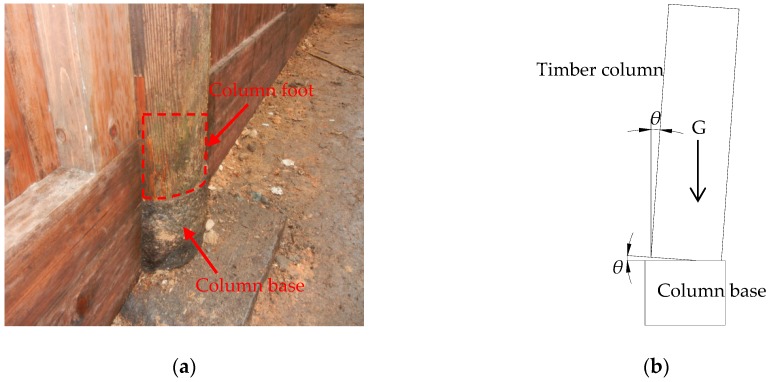
Connection type of Chinese traditional timber structure base: (**a**) Flat pendulum floating column foot; (**b**) A simplified model of tilted timber column.

The column foot in a Chinese traditional timber building is separated from the column base as shown in Figure 4. When the external moment is larger than the anti-overturning moment, the column rotates around the column base. Ignoring the bending deformation of timber column [27,28], the relative rotation *θ* of the column base and column foot as shown in Figure 4b, is equal to the inclination angle of the tilting timber structure (rigid rotation assumption).

**Figure 5:**
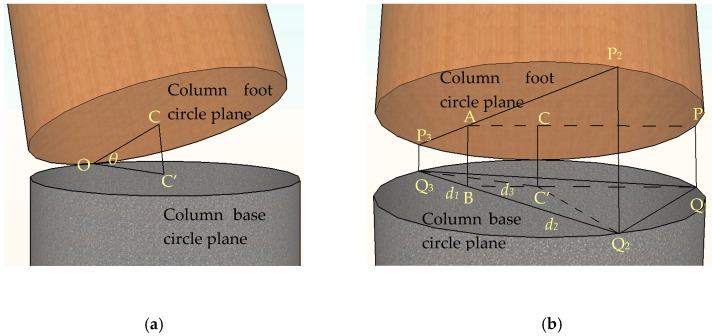
Analytical sketch of inclination angle: (**a**) Calculation diagram of inclination angle *θ*; (**b**) Calculation diagram of *l*_CC’_.

As is shown in Figure 5b, three measure points P_1_, P_2_ and P_3_ are randomly defined in the column foot circle plane. Correspondingly, three measurement points Q_1_, Q_2_ and Q_3_ are positioned in the same direction on the column base circle plane. The process is as follows:

(1) Connect from point Q_1_ to C’ and then extend to point B, which lies on the line Q_2_Q_3_ in the column base circle plane. Connect from point P_1_ to C and then extend to point A which is located on the line P_2_P_3_ in the column foot circle plane.

(2) On the Q_1_Q_2_Q_3_ plane, the length of Q_3_B (*d*_1_), Q_2_B (*d*_2_), BC’ (*d*_3_) can be calculated according to trigonometric analysis. And the length of Q_1_C’ is equal to the radius *r*.

(3) On the trapezoidal plane P_2_Q_2_P_3_Q_3_, the length of AB (*l*_AB_) can be calculated from the length of P_2_Q_2_ (*l*_2_) and the length of P_3_Q_3_ (*l*_3_) according to the proportional relationship as shown in Equation (7):

(7) $$l_{\mathrm{AB}}=\frac{d_{1}\cdot l_{2}+d_{2}\cdot l_{3}}{d_{1}+d_{2}}$$

(4) On the ABQ_1_P_1_ plane, the length of CC′ *l*_CC′_ can be calculated from the length of AB (*l*_AB_) and the length of P_1_Q_1_ (*l*_1_)

(8) $$l_{{\mathrm{CC}}^{\prime }}=\frac{d_{3}\cdot l_{1}+r\cdot l_{\mathrm{AB}}}{d_{3}+r}$$

**Figure 6:**
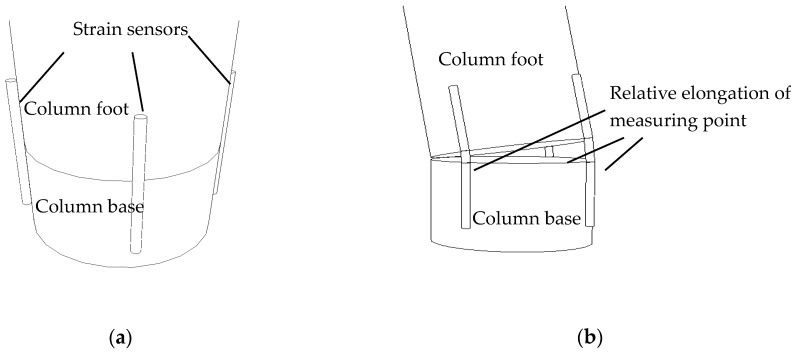
Layout of sensors: (**a**) Vertical column; (**b**) Inclined column.

(5) On the triangle OCC’ plane, the angle *θ* can be obtained through the cosine theorem, as is depicted in Equation (9):

(9) $$\cos\theta =1-\frac{{l_{{\mathrm{CC}}^{\prime }}}^{2}}{2r^{2}}$$

where, the length of *l*_1_, *l*_2_, and *l*_3_ can be measured by FBG sensors installed on the outer surface of the structure as shown in Figure 6.

In actual projects, when the timber column inclines, the FBG sensor will show a strain reading $\varepsilon_{i}$ (*i* = 1, 2, 3), and thus the *l_i_* (*i* = 1, 2, 3) can be calculated with Equation (10):

(10) $$l_{i}=\varepsilon_{i}\lambda_{i}$$

where $\lambda_{i}$ is the gauge length of the FBG sensor, *i* = 1, 2, 3.

#### 2.2.2. Applicability of Timber Column Monitoring Methods

This section will discuss the deployment strategy and the measurement range of the FBG sensors.

##### Deployment of Sensors

**Figure 7:**
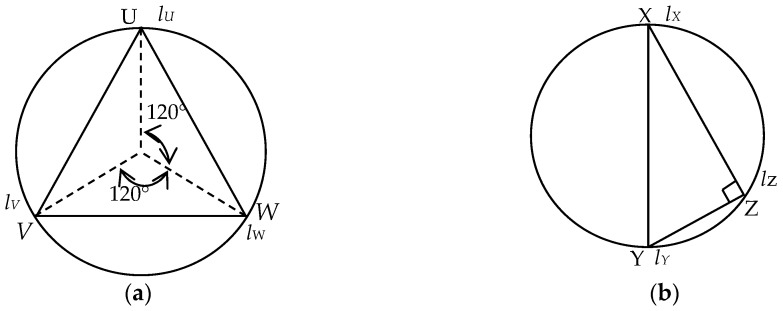
Layout of measuring points under special condition: (**a**) Equilateral triangle; (**b**) Right triangle.

The locations of three selected points are discussed. Theoretically, the sensors are randomly deployed. Herein we list two special conditions: (1) three measurement points make an equilateral triangle as shown in Figure 7a; (2) three measurement points make a right triangle as shown in Figure 7b. Under special condition (1) we define the relative lengths of the sensors as *l_U_*, *l_V_* and *l_W_*, respectively; while under special condition (2), the relative lengths of the sensors are defined as *l_x_*, *l_y_* and *l_z_*, respectively.

Under special conditions (1) or (2), Equation (8) can be reduced to Equations (11) or (12), respectively, which makes it simple and convenient to solve the inclination angle:

(11) $$l_{{\mathrm{CC}}^{\prime }}=\frac{l_{U}+l_{V}+l_{W}}{3}$$

(12) $$l_{{\mathrm{CC}}^{\prime }}=\frac{l_{X}+l_{Y}}{2}$$

When it comes to the special condition (2), however, the *l*_CC’_ is calculated by using only two sensors (sensor X and sensor Y) as shown in Figure 7b. Compared with using three sensors under normal condition, a larger error is spotted with two sensors. Thus, in actual projects, we should avoid the case, in which three sensors make a right triangle as shown in Figure 7b.

##### Applicable Range

The current specification [8] specifies that the allowable limit value of the lateral displacement does not exceed *H*/120 (*H* is the height of column), i.e., the maximum inclination angle of timber column is 0.48°. When the rotation center point O, the column base plane center point C’, and one of three FBG sensors are collinear, we obtain the most unfavorable layout of measurement points. Then the Equation (8) can be transformed into the Equation (13). Under this condition, the strain growth rate of the sensor is the fastest, which causes the sensor to preferentially reach its measurement limit and reduces the measurement range.

(13) $$l_{{\mathrm{CC}}^{\prime }}=\frac{l_{m}}{2}$$

where *l_m_* is the FBG sensor’s deformation at the most unfavorable position, *m* = X, Y, Z.

Substituting Equation (13) into Equation (9), it can get:

(14) $$1-\cos(0.48{}^{\circ})\leq \frac{{l_{{\mathrm{CC}}^{\prime }}}^{2}}{2r^{2}}=\frac{{\left(l_{m}/2\right)}^{2}}{2r^{2}}=\frac{{\left(\lambda_{m}\varepsilon_{m}/2\right)}^{2}}{2r^{2}}$$

It can be seen from Equation (14) that the longer the gauge length of sensor is, the larger the range of inclination angle is. In general, the extreme strain of FBG sensor $\varepsilon_{m}$ is 7000 uε. Therefore, the relationship between the gauge length of FBG and the radius of measured timber column can be calculated through Equation (14) as shown in Table 2.

### 2.3. Dislocation Monitoring of Mortise-Tenon Joints

The detachment of tenon from the mortise mouth is a unique failure mode for the Chinese traditional timber buildings. The damage level of the mortise-tenon joint is defined by the amount of the detachment [8]. Therefore, the monitoring of the amount of dislocation is also an important part of the deformation monitoring for Chinese traditional buildings in service.

**Figure 8:**
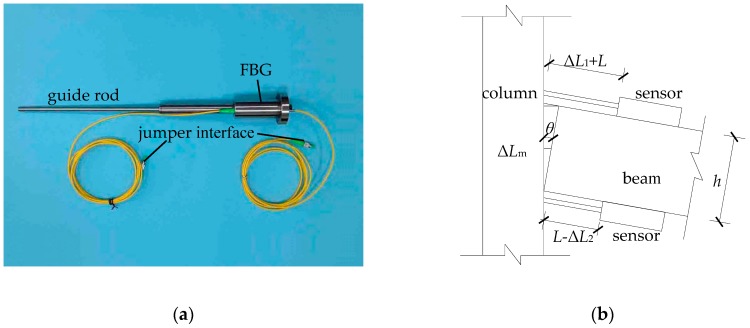
(**a**) FBG displacement sensor; (**b**) Sensor arrangement.

This section analyzes how to use the FBG displacement sensor to monitor the amount of dislocation for the mortise-tenon joint as shown in Figure 8a, whose accuracy is as high as 0.1 mm, and the measurement range is 50 mm–100 mm, which can fully meet the requirements of measurement accuracy and maximum dislocation measurement range.

Here *L* is the original length of the guide rod and *h* is the height of the beam section. When the beam or the column rotates or slips as a result of external forces, the slip amount of the tenon away from the mortise mouth is defined as Δ*L*_1_, and on the opposite side of the joint the inset amount is defined as Δ*L*_2_. The relative rotation angle between beam and column is *θ*. At this time, the sensor reading on the pull side is *L* + Δ*L*_1_, and the other side is *L* − Δ*L*_2_. Considering the fact that the slippage has positive and negative properties, the relative slippage Δ*L_m_* along the longitudinal axis of the beam is taken as the actual amount of dislocation. From the observation in Figure 8b, Δ*L_m_* can be easily obtained using the following Equation (15):

(15) $$\Delta L_{m}=\frac{\cos\theta \cdot (\Delta L_{1}-\Delta L_{2})}{2}$$

It means that the tenon of beam moves away from the mortise mouth of the column when Δ*L_m_* is positive, while it moves towards the mortise mouth of the column when Δ*L_m_* is negative. Considering that the inclination angle of the column is approximately equal to the relative rotation angle between the beam and column, *θ* in the above equation can be obtained from the inclination angle of the column in Section 2.2.

## 3. Experimental Validation

Experiments were conducted to verify the effectiveness of the proposed deformation monitoring methods of timber structure from three aspects, i.e. beam, column and mortise-tenon joint, respectively.

### 3.1. Deflection Monitoring of Timber Beam

#### 3.1.1. Test Setup and Sensors Placement

**Figure 9:**
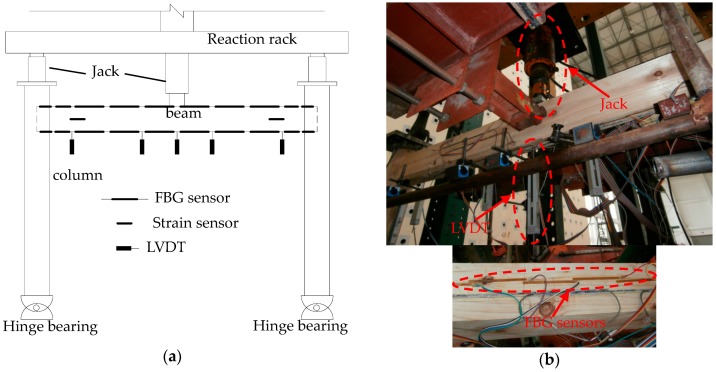
Test setup and layout of sensors: (**a**) Setup and layout of measurement points; (**b**) Photos of sensor layout.

A Tousun timber frame model was made with the second-grade hall log [26] at a scale ratio of 1:3.52 as shown in Figure 9, and the length of the beam *L* is 2.4 m, column’s height *H* is 1.8 m, and beam’s height *z* is 120 mm. The span-to-height ratio *k* is 20. Loads of 10 kN were applied on the midspan of beam and the two tops of the columns with jacks. The timber beam was divided into 8 units and each unit is 300 mm long. Two sides of the anchorage end for FBG sensors were affixed on the middle of the upper and lower surfaces of each unit for the timber beam. LVDTs were mounted at the midspan of the beam and both ends of the timber beam to measure the deflection of the beam.

FBGs [29] with a gauge length of 300 mm are used during all experiments in this article. And they were mounted on the structure are as follows: (1) Clean the dust on the wooden beam to be tested; (2) Apply epoxy resin on the surface of the beam, where FBG sensors are mounted and keep away from wood knots and small cracks on the beam; (3) Attach the FBG to the beam as required.

#### 3.1.2. Timber Beam Test Data and Analysis

Table 3 shows the settlement values measured at both ends of the timber beam under each load level. The absolute values of the strain difference Δε between the upper and lower surfaces measured by the FBG sensors are shown in Table 4. 

Take the mid-span flexural deflection as an example to illustrate the specific calculation process. Herein, the subscript of midspan node *i* is 4. By comparing the right terms of Equations (4) and (6), the deflection in midspan of beam under different load levels can be obtained as follows:

(16) $$\begin{array}{ll} \omega_{4}= & {\left(300\right)}^{2}\left[\begin{array}{l} 0.5\times \left(\begin{array}{l} \frac{7.5\varepsilon_{1}}{z_{1}}+\frac{6.5\varepsilon_{2}}{z_{2}}+\frac{5.5\varepsilon_{3}}{z_{3}}+\frac{4.5\varepsilon_{4}}{z_{4}}+\frac{3.5\varepsilon_{5}}{z_{5}}\\ +\frac{2.5\varepsilon_{6}}{z_{6}}+\frac{1.5\varepsilon_{7}}{z_{7}}+\frac{0.5\varepsilon_{8}}{z_{8}} \end{array}\right)\\ -(3.5(\frac{\varepsilon 1}{z_{1}})+2.5(\frac{\varepsilon_{2}}{z_{2}})+1.5(\frac{\varepsilon_{3}}{z_{3}})+0.5(\frac{\varepsilon_{4}}{z_{4}})) \end{array}\right]\\ & +0.5\Delta_{l}+0.5\Delta_{r} \end{array}$$

where $\omega_{4}$ is the deflection deformation value at the demarcation points between units 4 and 5, i.e., the midspan node.

**Figure 10:**
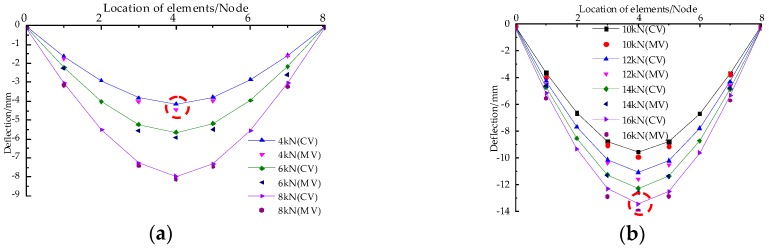
A comparison between MV and CV under different load levels: (**a**) Deflections with load levels 4 kN to 8 kN; (**b**) Deflections with load levels 10 kN to 16 kN.

Figure 10 shows the comparison between the measured value (MV) read from the LVDT and the calculated value (CV) derived from the FBG readings using the method proposed in this paper. It can be seen from the above figure that a good agreement exists between the MV and CV along the whole span of the beam under different load levels. At the initial stage of loading (F = 4 kN), the relative error between the CV and MV is 8.2% at the maximum. As the load increases, the displacements obtained by FBG monitoring tend to be consistent with the measured displacements from three linear variable differential transducers (LVDTs). When the mid-span displacement reaches 13 mm, the relative error between MV and CV is 5.7%. This indicates that the method proposed in the paper has a high precision in the deformation monitoring. The reasons for the relative errors are twofold: (1) there exists a small gap between test instrument and tested beam, thus resulting in difference of readings from LVDTs and the FBG sensors at the beginning of loading; and (2) the Euler Beam Theory doesn’t take into account the effect of shear deformation on strain.

The greater the beam’s span-to-height ratio is, the smaller the effect of the shear deformation is, and as a result, the accuracy of the proposed method also becomes increasingly higher. Considering the fact that the *k* of the most Chinese traditional timber beams in service ranges from 20 to 40, it can be seen that this method proposed in this paper can be applicable to monitoring deformation of Chinese traditional timber beams.

### 3.2. Tilting Deformation Monitoring of Timber Column

#### 3.2.1. Test Setup and Sensors Placement

**Figure 11:**
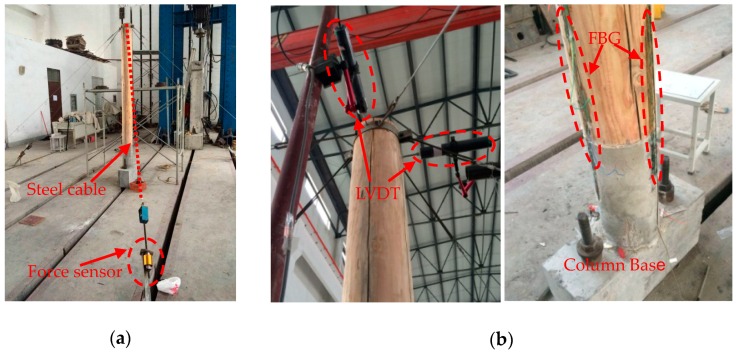
Test setup: (**a**) Overall photo of a single-column pushover test; (**b**) Layout of sensors on the top and foot of the column.

In this section, a single-column pushover test was conducted in the laboratory as shown in Figure 11. The column diameter is 200 mm and the column height is 2000 mm. Six pre-tensioned steel cables with springs were used to exert axial force on the top of the column and maintain the stability of the column. Each cable was connected with a force sensor to easily collect the tension of each cable at any time. 

**Figure 12:**
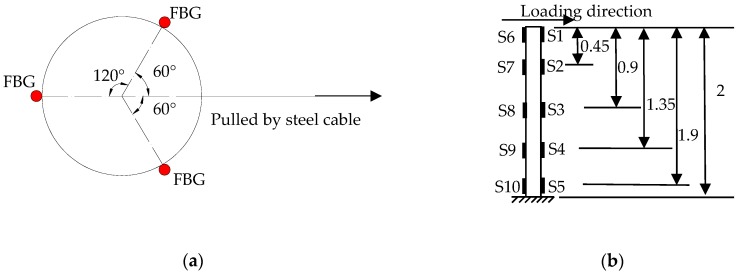
Layout of sensors: (**a**) FBG sensors; (**b**) Strain gauges (unit: m).

Three long-gauge FBG strain sensors were attached to the column base and column foot surfaces after the pre-tightening force was applied. The FBG’s installation procedure on the column is similar to that on the beam. During loading stage, a steel cable needs to be tilted to gradually apply the traction force of the corresponding direction and make the column body slowly tilt. Three FBGs with a gauge length of 300 mm were equilaterally mounted on the plane of the pillar foundation as shown in Figure 12a. To protect those sensors, the test was halted once the FBG tensile strain approached 6000 uε. The strain gauges were evenly distributed at an interval of 40 cm on both sides of the sloping column so as to analyze column body’s deformation, as shown in Figure 12b. To measure the tilting amount of the column, four LVDTs were mounted on the top and middle of the column along the two directions that are parallel to and vertical to the inclined column, respectively. Figure 11b shows the layout of four LVDTs on the top of the column. The weighted average of the horizontal displacements in both directions was used to analyze the maximum inclination angle of the column.

#### 3.2.2. Tilt Test Process and Results Analysis

##### Calculation and Analysis of Column Inclination 

The test involves two stages: (1) loading stage; (2) unloading stage. The strain increment is 500 uε for FBG sensors. Test goes into the unloading stage once the strain value reaches 6000 uε for any FBG sensors. Table 5 and Table 6 show the measured strain values of the three FBG sensors in the stages of the test loading and unloading, respectively. 

**Figure 13:**
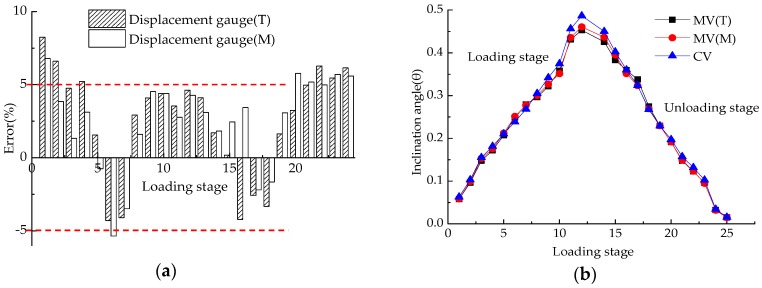
Comparison of error and inclination angle: (**a**) Errors between experiment values from the top LVDTs or middle LVDTs and calculated value from FBGs; (**b**) Inclination angles.

Since the layout of three sensors takes a shape of an equilateral triangle, the strain values of FBG in Table 5 and Table 6 are substituted into Equation (10) to obtain *l_U_*, *l_V_*, *l_W_*, which are then substituted into Equation (11) to get *l*_CC’_. Afterwards, substitute *l*_CC’_ into Equation (9) to obtain the inclination angle *θ*. Figure 13 shows the comparison results using different methods at different loading levels. Here MV and CV represent the measured value and calculated value of inclination angle, respectively. The T and M represent the measured value of LVDTs mounted on the top and middle of the column, respectively. Generally speaking, from Figure 13a, it can be seen that both CV and MV(T) or MV(M) are in good agreement and that the error between the actual measured value and the calculated value fluctuates by about 5%, which indicates better accuracy with the FBG-based tilt monitoring method.

The relative error of 5% may be contributed to the small deformation measurement in this conducted experiment for both the instrument and test operation (less than 0.5°), of a very high precision. However, it is difficult for the traditional electronic LVDTs to achieve such high accuracy when it is used to measure small deformation.

##### Verification of Rigid Rotation for the Column 

**Figure 14:**
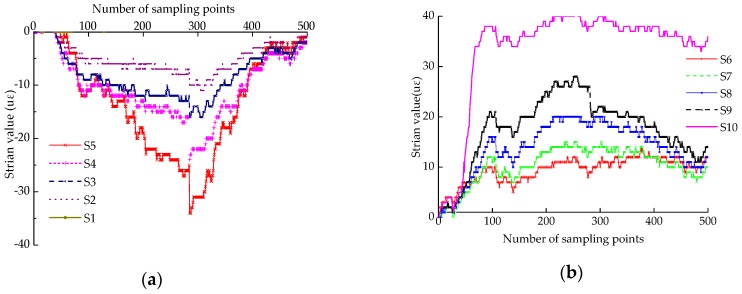
Strain values of the column’s body: (**a**) Measurement points S1–S5; (**b**) Measurement points S6–S10.

According to Figure 13b, it can be seen that the inclination angles measured by the LVDTs on the top and middle of the column are almost the same, of rigid rotation during the test. In addition, the strain values of the column body were also measured by 10 strain gauges laid out as shown in Figure 12b during the test process and are shown in Figure 14. 

It can be seen that the strain values in the loading stage are in the range of ±40 uε, which proves that the deformation of the column itself is very small when subjected to external forces and can be considered negligible compared with the overall deformation. Furthermore, this indicates that the column almost approaches rigid body rotation when it tilts. The conclusion is consistent with the results from the collapse test of the overall timber frame under external forces [28].

### 3.3. Test Verification: Dislocation Monitoring of Mortise-tenon Joint

This experiment adopted an electronic LVDT to replace the FBG displacement sensor to monitor the dislocation of mortise-tenon joint. The reasons for this are twofold: (1) both the FBG displacement sensors and the traditional electronic LVDTs used to monitor the displacement are designed under similar measuring principles [30,31], to be specific, the displacement amount of the guide rod can be converted into a magnitude that reflects the displacement of the measured target; and (2) Some of FBG sensors are faulty during the experiment and the experiment has to be conducted with LVDTs instead.

#### Test Setup and Layout of Sensors

**Figure 15:**
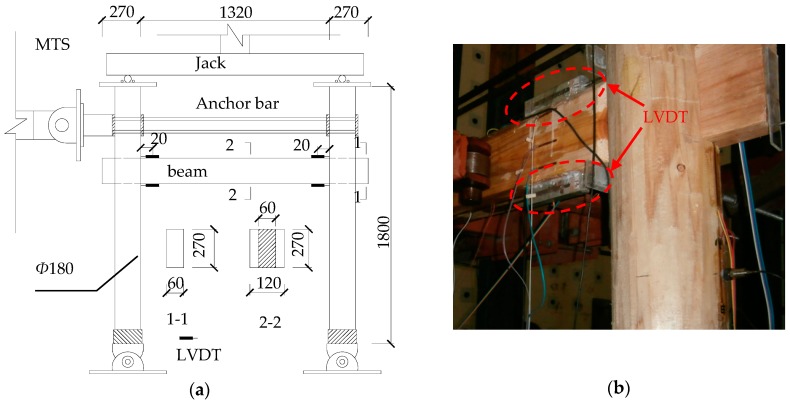
Test setup and sensor layout: (**a**) Timber frame loading setup; (**b**) LVDT layout.

The structural model and the test setup are shown in Figure 15. Two LVDTs were arranged on the top and bottom surfaces of each beam end as shown in Figure 15b. The MTS actuator was mounted on the column cap with an anchor and applied a low-cycle repeated load to the timber frame. The displacement increment was 20 mm for each stage, and each cycle was repeated three times.

Substitute the measurement data of LVDTs into Equation (15), so the amount of dislocation at each loading amplitude can be calculated as shown in Table 7.

**Figure 16:**
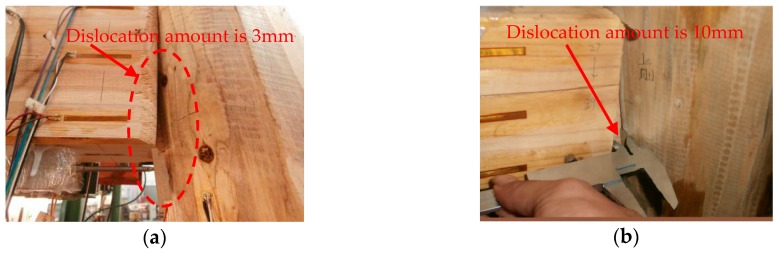
Measured dislocation with a: (**a**) loading amplitude of 80 mm; (**b**) loading amplitude of 160 mm.

It can be seen that the cumulative dislocation amount gradually increases with the increase of the loading amplitude, and the amount of dislocation on both sides is basically the same. When the frame has the largest lateral displacement, the amount of dislocation can reach 10 mm for the joint. By comparing the dislocations measured by a Vernier caliper on the spot (Figure 16) with the data from Equation (15) in Table 7, it can be seen that the results are consistent. Due to the limitation of the experimental conditions, the electronic LVDTs rather than optical LVDTs were used to measure the amount of dislocation in the experiment and the experimental results look ideal. This indicates that the dislocation monitoring method is feasible and effective.

## 4. Conclusions 

This paper proposes deformation monitoring methods based on FBG sensing, which can be applicable to monitoring beam deflection, column inclination angle and mortise-tenon joint dislocation for Chinese traditional timber structures. A series of experiments were conducted to verify the applicability and effectiveness of the proposed algorithms. The concluding remarks are drawn as follows:

(1) This paper proposes a timber beam deflection monitoring method that takes account of semi-rigid joint support, support settlement and irregular section, which is applicable and proved effective for Chinese traditional timber beams through experimental validation. The precision of the algorithm is dependent on the beam’s span-to-height ratio and the number of the sensors.
(2) The FBG-based timber column tilt monitoring technology is applicable and effective by comparing the timber column tilt amount from the LVDTs and the method proposed in this paper. Besides, it can be drawn that the column rotates rigidly when it tilts through comparing the column strain measured by the strain gauge and the amount of column tilt.
(3) The dislocation monitoring method is proposed to monitor the mortise-tenon joints for Chinese traditional buildings. The applicability and effectiveness are indirectly validated by experimental results.

The effectiveness of the deformation monitoring methods proposed in this paper is validated by a limited number of experimental results. More experiments and numerical simulation are to be conducted in the future. More influencing factors are also needed to be identified for the deformation monitoring methods. It is believed that more and more monitoring techniques based on FBG sensing will be developed and applied to practical engineering with the depth of research.

## Figures and Tables

**Table 1:** The optimum number of measuring points.

Span-To-Height Ratio *k*		*k* ≥ 30	25 ≤ *k* < 30	20 ≤ *k* < 25
Length of span	<5 m	6	8	8
	≥5 m	6	8	10

**Table 2:** Maximum radius of timber column using different gauge length of FBG sensors (Unit: mm).

Gauge Length *l*	100	200	300	400	500	600	700	800	1,000
Radius *r*	42	84	125	167	201	251	292	334	418

**Table 3:** Settlement values at beam ends of timber frame model under different load levels.

Load Level *F*/kN	4	6	8	10	12	14	16
Δ*_l_*/mm	−0.06	−0.1	−0.12	−0.16	−0.22	−0.28	−0.32
Δ*_r_*/mm	−0.02	−0.06	−0.1	−0.14	−0.2	−0.26	−0.28

**Table 4:** Strain difference between the upper and lower surfaces for each element.

d ^1^ /mm	Strain Difference under Different Loads/με						
	4 kN	6 kN	8 kN	10 kN	12 kN	14 kN	16 kN
150	190	300	367	421	457	503	538
450	440	579	714	846	993	1067	1137
750	676	941	1242	1510	1707	1900	2058
1050	849	1222	1633	2015	2361	2590	2797
1350	929	1137	1883	2118	2404	2599	2833
1650	607	877	1169	1454	1749	2034	2319
1950	438	599	783	953	1120	1266	1406
2250	190	291	361	420	480	545	587

^1^ The distance from the midpoint of each element to the left endpoint of the beam.

**Table 5:** Strain values of three FBG sensors in loading stage.

No. of Sensors	Strain under Different Loading Stages/uε											
	1	2	3	4	5	6	7	8	9	10	11	12
1	531	964	1654	1995	2432	2927	3560	4023	4461	4852	5852	6261
2	346	531	730	830	919	1008	1117	1174	1231	1259	1330	1335
3	217	303	322	341	322	227	4	127	276	426	783	896

**Table 6:** Strain values of three FBG sensors in the unloading stage.

No. of Sensors	Strain under Different Loading Stages/uε											
	13	14	15	16	17	18	19	20	21	22	23	24
1	5754	5111	4532	4033	3383	2797	2208	1628	1328	924	268	124
2	1339	1319	1320	1286	1188	1080	938	805	710	545	201	109
3	762	591	432	325	100	123	297	311	264	320	126	43

**Table 7:** Amount of dislocation at each loading amplitude (Unit: mm).

Loading Amplitude	20 ^1^	−20 ^2^	40	−40	60	−60	80	−80
Left column	−0.066	0.044	−1.749	1.379	−1.991	2.45	−2.255	3.043
Right column	−0.165	−0.385	−0.55	−1.309	1.705	−2.046	0.583	−4.07
Loading Amplitude	100	−100	120	−120	140	−140	160	−160
Left column	−5.175	3.7125	−7.5	4.4875	−8.575	6.096	−8.062	8.695
Right column	4.68	−2.9	5.985	−4.05	7.2325	−6.412	9.390	−7.5

^1,2^ Positive values indicate pressure and negative values indicate tension in the above table.

## References

[1] Kan Y., Zhao H.T., Xue J.Y., Sui Y. (2008). Monolithic stability analysis of timber frame in historical buildings. World Earthqu. Eng..

[2] Lorenzoni F., Casarin F., Modena C., Mauro C., Kleidi I., Francesca P. (2013). Structural health monitoring of the Roman Arena of Verona. J. Civ. Struct. Health Monit..

[3] Min K.W., Kim J., Park S.A., Park C. (2013). Ambient vibration testing for story stiffness estimation of a heritage timber building. Sci. World J..

[4] Wu M.H. (2017). Study on Monitoring Technique and Damage Evolution Method of Ancient Timber Structure Considering the Long-Term Environment Effect and Seismic Effect. Ph.D. Thesis.

[5] Fang D.P., Iwasaki S., Yu M.H., Shen Q.P., Miyamoto Y., Hikosaka H. (2017). Ancient Chinese timber architecture. II: dynamic characteristics. J. Struct. Eng..

[6] Hanhifirvi A. (2000). Computational method for predicting the long-term performance of timber beams in variable climates. Mater. Struct..

[7] Risia A.D., Focacci F., Luciano R. (2014). Experimental investigation on flexural behavior of timber beams repaired with CFRP plates. Compos. Struct..

[8] Liang T., Wang Y.W., Ni T.Z. (1992). GB/50165-1992. Technical Specification for Maintenance and Reinforcement of Ancient Buildings.

[9] Luo M., Li W., Wang J., Wang N., Chen X., Song G. (2018). Development of a novel guided wave generation system using a giant magnetostrictive actuator for nondestructive evaluation. Sensors.

[10] Lu G., Feng Q., Li Y., Wang H., Song G. (2017). Characterization of ultrasound energy diffusion due to small-size damage on an aluminum plate using piezoceramic transducers. Sensors.

[11] Ho S.C.M., Li W., Razavi M., Song G. (2017). Fiber bragg grating based arterial localization device. Smart Mater. Struct..

[12] Qi B., Kong Q., Qian H., Patil D., Lim I., Li M., Liu D., Song G. (2018). Study of impact damage in PVA-ECC beam under low-velocity impact loading using piezoceramic transducers and PVDF thin-film transducers. Sensors.

[13] Dumoulin C., Deraemaeker A. (2017). Real-time fast ultrasonic monitoring of concrete cracking using embedded piezoelectric transducers. Smart Mater. Struct..

[14] Song G., Li W., Wang B., Ho S.C.M. (2017). A review of rock bolt monitoring using smart sensors. Sensors.

[15] Todd M., Yeager M., Key C., Gregory W. (2017). Assessment of embedded Fiber Bragg Gratings for structural health monitoring of composites. Struct. Health Monit..

[16] Li W., Xu C., Ho S.C.M., Wang B., Song G. (2017). Monitoring concrete deterioration due to reinforcement corrosion by integrating acoustic emission and FBG strain measurements. Sensors.

[17] Mao J., Xu F., Qian G., Liu S., Jin W., Xu Y. (2016). A monitoring method based on FBG for concrete corrosion cracking. Sensors.

[18] Jiang S.F., Tang W.J., Wu M.H. (2016). Deformation monitoring method of ancient wooden beam based on FBG strain measurement. Earthqu. Eng. Eng. Vibr..

[19] Feio A., Machado J.S. (2015). In-situ assessment of timber structural members: Combining information from visual strength grading and NDT/SDT methods—A review. Constr. Build. Mater..

[20] Li H.N., Li D.S., Song G.B. (2004). Recent applications of fiber optic sensors to health monitoring in civil engineering. Eng. Struct..

[21] Marsili R., Rossi G., Speranzini E. (2018). Fibre Bragg Gratings for the monitoring of wooden structures. Materials.

[22] Wang J., Yang N., Yang Q.S. (2015). Structural health monitoring system for heritage buildings. J. Beijing Jiaotong Univ..

[23] Shen S., Wu Z.S., Yang C.Q. (2010). Research on Monitoring Deformation Distribution of Structure by Improved Conjugate Beam Method Based on Distributed Optical Fiber Strain Sensing Technology. China Civ. Eng. J..

[24] Sun X.F., Fang X.S., Guan L.T., Hu Z.Q., Guo L., Jiang X.Y. (2012). Material Mechanics I.

[25] Li J. (1933). Chinese Ancient Building Method.

[26] Wang T. (1992). Research on the Static Performance of Chinese Ancient Wood Frames.

[27] Wu M.H., Tang W.J., Jiang S.F. (2018). Deformation Monitoring Strategy of Ancient Built Wood Structure Based on Distributed Optical Fiber Technology. J. Fuzhou Univ..

[28] Zhao H.T., Zhang X.C., Xue J.Y. (2011). Conceptual design thoughts of Chinese ancient timber buildings. J. Xi’an Univ. Archit. Technol..

[29] Li S.Z., Wu Z.S. (2007). Development of Distributed Long-gauge Fiber Optic Sensing System for Structural Health Monitoring. Struct. Health Monit..

[30] Ren L. (2008). Application of Fiber Grating Sensing Technology in Structural Health Monitoring. Ph.D. Thesis.

[31] YHD-Displacement Sensor YDH DC Displacement Sensor. https://wenku.baidu.com/view/aff51c3a83c4bb4cf7ecd160.html.

